# Teleradiology for remote consultation using iPad improves the use of health system human resources for paediatric fractures: prospective controlled study in a tertiary care hospital in Italy

**DOI:** 10.1186/1472-6963-14-327

**Published:** 2014-07-28

**Authors:** Floriana Zennaro, Daniele Grosso, Riccardo Fascetta, Marta Marini, Luca Odoni, Valentina Di Carlo, Daniela Dibello, Francesca Vittoria, Marzia Lazzerini

**Affiliations:** 1 Departments of Radiology, Institute for Maternal and Child Health IRCCS Burlo Garofolo, Trieste, Italy; 2 Unit Of Research on Health Services and International Health, Institute for Maternal and Child Health IRCCS Burlo Garofolo, Trieste, Italy; 3 Departments of Orthopaedics, Institute for Maternal and Child Health IRCCS Burlo Garofolo, Trieste, Italy

**Keywords:** Teleradiology, Children, Fractures, Organisation of care, Cost

## Abstract

**Background:**

The growing cost of health care and lack of specialised staff have set e-Health high on the European political agenda. In a prospective study we evaluated the effect of providing images for remote consultation through an iPad on the number of in-hospital orthopaedic consultations for children with bone fractures.

**Methods:**

Children from 0 to 18 years diagnosed with a bone fracture by the radiologist during the hours when an orthopaedic service is provided only on-call were eligible for enrollment. Cases were enrolled prospectively during September and October 2013. A standard approach (verbal information only, no X-Ray provided remotely) was compared to an experimental approach (standard approach *plus* the provision of X-ray for remote consultation through an iPad). The primary outcome was the number of orthopaedic in-hospital consultations that occurred. Other outcomes included: immediate activation of other services; time needed for decision-making; technical difficulties; quality of images and diagnostic confidence (on a likert scale of 1 to 10).

**Results:**

Forty-two children were enrolled in the study. Number of in-hospital consultancies dropped from 32/42 (76.1%) when no X-ray was provided to 16/42 (38%) when the X-rays was provided (p < 0.001). With remote X-ray consultation in 14/42 (33.3%) cases services such as surgery and plaster room could be immediately activated, compared to no service activated without teleradiology (p < 0.001). Average time for decision making was 23.4 ± 21.8 minutes with remote X-ray consultation, compared to 56.2 ± 16.1 when the X-ray was not provided (p < 0.001). The comparison between images on the iPad and on the standard system for X- Ray visualisation resulted in a non statistically significant difference in the quality of images (average score 9.89 ± 0.37 vs 9.91 ± 0.30; p =0.79), and in non statistically significant difference in diagnostic confidence (average score 9.91 ± 0.32 vs 9.92 ± 0.31; p = 0.88).

**Conclusions:**

Remote X-ray consultation through Aycan OsiriX PRO and iPad should be considered as a means for reducing the need of in-hospital orthopaedic consultation during on-call times, and potentially decrease the cost of care for the health system. In the future, alternative systems less expensive than Aycan OsiriX PRO should be further developed and tested.

## Background

European health systems are under severe budgetary constraints, while having to respond to the challenges of an ageing population, rising expectations of citizens, and a steady decline in the number of health personnel [1,2]. All these factors have set e-Health high on the European political agenda: the *eHealth Action Plan 2012–2020 - Innovative healthcare for the 21st century*[2] is strengthening European commitment in the implementation of e-Health services since they are believed to have the potential to both reduce cost and improve the quality of health care. Due to the predominant digital character of medical imaging, radiology is on the forefront in this e-Health scene [3].

Injuries are one of the primary causes of morbidity in children [4,5]. According to existing epidemiological data, about a quarter of children suffer from at least one injury every year [4-6]. Incidence of fractures range from 2 to 3.6 per 100 children a year [4-7], with one-third of boys and girls expected to suffer from a fracture before 16 years of age [6]. Fractures also represent a considerable economic burden to families, with high health-care related costs and lost parental working time [8-10].

Most cases of fracture access hospital care through the emergency department. Based on the case characteristics and on existing local guidelines, the emergency department staff contact an orthopaedic specialist for support and advice. Usually, this remote support relies on a verbal description made by the emergency department staff or on a written description made by the radiologist. However, clinical practice has showed that a description of the X-ray is not enough for proper decision making in orthopaedics and often does not substitute for viewing the actual images [8,9]. This is particularly true for non-displaced or minimally displaced fractures [12].

When orthopaedic specialists lack the X-ray image for remote consultation they may decide to travel to the hospital to view the image, or decide to base their opinion solely on the X-ray description. The first option implies additional costs for the health services (cost of the orthopaedic consultancy), while the second option implies a possible risk of misdiagnosis and improper case-management [12,13].

Implementing teleradiology for remote X-ray consultation into standard clinical practice has the potential to improve this communication. Remote X-ray consultation could enable the on-call orthopaedic specialist to establish the management plan more accurately and more rapidly with a possible benefit both for the patient (speeding up of case-management) and for the health services (better use of health services resources).

Although a number of pilot experiences exist, and experiences are rapidly growing in this field, remote X-ray consultation on mobile devices for on-call specialist services in Europe is still not a wide-spread practice. Literature in orthopaedics has so far mostly concentrated on the use of digital cameras for acquiring a digital copy of the plain X-ray image, and on the use of either Multimedia Messaging Services (MMS) or image-transfer [11-14]. Studies were generally small in sample size, and MMS have in most instances failed to show an acceptable accuracy and reliability for diagnosis, with a misdiagnosis risk reported within the range of 15 to 40% [15-17]. Moreover, the use of either MMS or emails to transfer patients files is not complying with the international regulations and standards on data privacy and integrity, and may be open to litigation. The European legislation, similarly to the United states regulation, requires that the images are transferred securely and are accessed only by authenticated users [3,18,19].

Alternative methods for remote consultation are needed for optimising the use of resources in emergency departments. In this prospective study we evaluated the impact of remote X-ray consultation using a mobile device (iPad) on orthopaedic consultations for children with bone fractures in a tertiary care paediatric hospital in Italy.

## Methods

### Setting

The Institute for Maternal and Child Health IRCCS Burlo Garofolo is tertiary care center in Northern Italy. The emergency paediatric unit is open 24 hours a day, 7 days/week, and can be accessed by children and their families either by self referral or as referral by other doctors/hospitals. During 2013, the total number treated in the emergency department was 21,968 children. Of these, 3,505 (15.9%) children attended the unit because of an injury, and 3,415 (15.5%) orthopaedic consultancies were requested.

During the afternoons and week-ends (4 pm-8 am Monday to Friday, and from Saturday 1 pm to Monday 8 am) orthopaedic specialists are not on active service in-hospital but only available on-call. During these on-call hours, when a child is diagnosed with a bone fracture, the paediatrician in charge in the emergency department phones the orthopaedic on-call who is responsible for case management. The paediatrician describes to the orthopaedic specialist on-call the characteristics of the clinical case and the diagnosis as from the written report of the radiologist. Based on this information, the orthopaedic specialist on-call decides whether the case deserves an urgent management (in this case the orthopaedic specialist will need to come into the hospital), or whether the case can be referred to the following day when an orthopaedic specialist is working in the hospital. Before this current study there was no routine system for providing the X-ray images to the orthopaedic specialist on-call for remote consultation.

### Ethical approval

The study was approved by the Ethical Review Board of the IRCCS Burlo Garofolo. All X-Ray images were obtained as a part of the routine health care, for diagnostic purposes based on the child’s presenting complaint. All the children and their parents gave written consent to the radiology examinations. The IRCCS Burlo Garofolo review board did not require a specific written consent for teleradiology, based on the following considerations: a) all patients treated at IRCCS Burlo need to sign a written consent for all diagnostic procedures including radiology, and specific information on the use of teleradiology services is provided to all patients; b) the radiological and orthopaedic examinations were performed as a part of routine care; c) teleradiology at Burlo is based on a secured and accredited system, protecting data privacy.

### Equipment

The equipment consisted of: i) an iPad Retina display; ii) Aycan OsiriX PRO, an App for transferring and displaying DICOM images on the iPad; iii) a Mac computer, connected with the PCAS (Picture Archiving and Communications System) of IRCCS Burlo Garofolo.

Aycan OsiriX PRO is built on Macintosh's operating system. Among the applications for the visualisation of images available on the market, Aycan was chosen because it can easily integrate into any DICOM workflow, it is easy to use, and at time of the study design it was one of the few systems fully accredited for remote visualisation of radiological images. Information security regarding confidentiality, integrity, availability and accountability is ensured and complies with the current regulatory standards. The Aycan mobile App is compliant with DICOM 3.0 standards and ISO 13485 standards, and is labelled with a CE-Mark as a Medical Device Class I in Europe and with FDA 510 (k) clearance in the USA, which ensures full conformity with existing regulations. The display of the iPad is a 9.7" touch-screen with Retina display resolution (2.048 × 1.536 pixels at 264 pixels per inch (ppi))reaches regulations of DIN V 6868–57:2001–02 (Consistency and uniformity testing for medical displays) [20,21].

**Figure 1:**
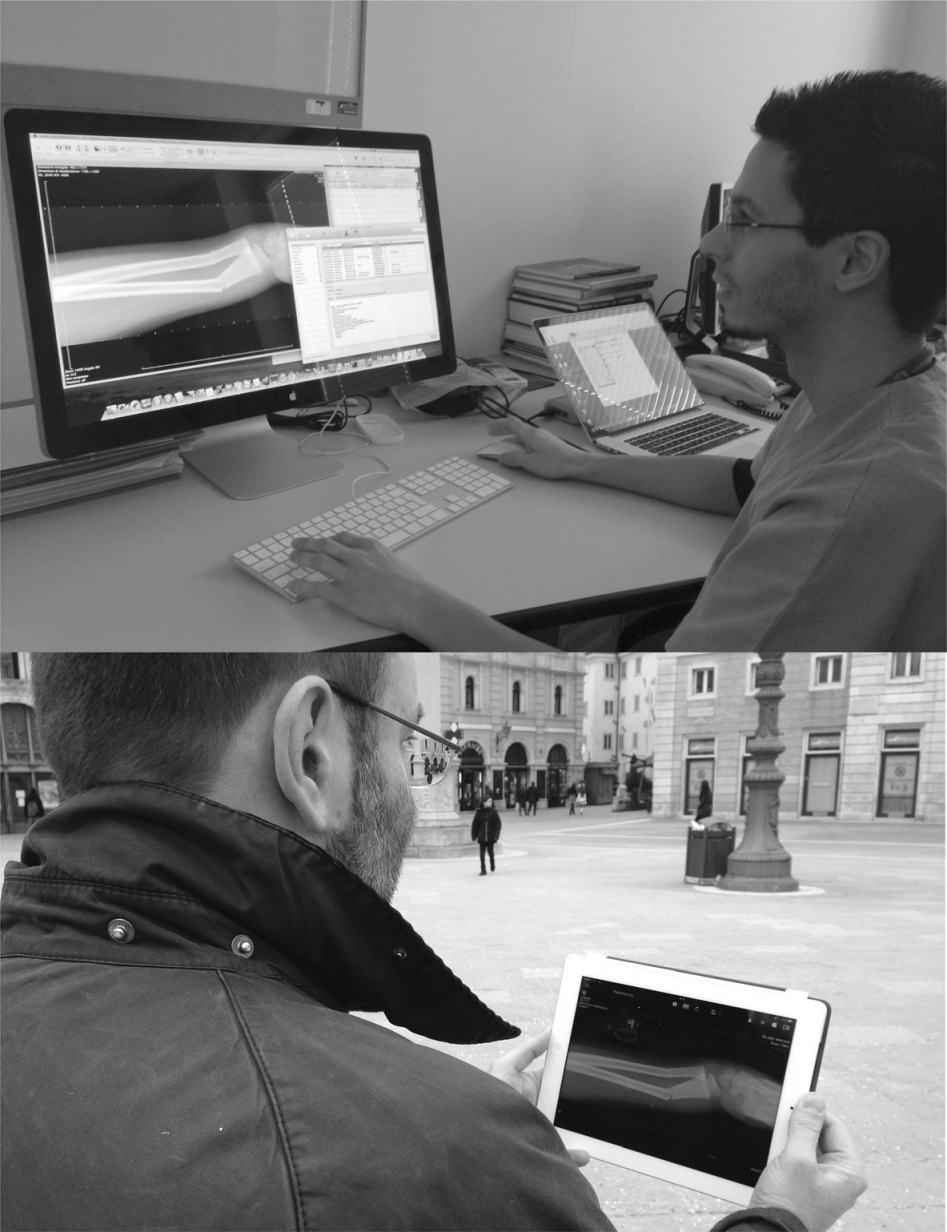
**Data transfer from in-hospital PACS workstation and the iPad for remote consultation.** Example of process.

With Aycan OsiriX PRO the DICOM images are stacked into cases (at the Aycan OsiriX PRO software inside the hospital/imaging center) and sent to iPads. Users have to setup a login at the mobile.aycan.com server. This server does not store any patient data and is only used for establishing the proper connection between sender and receiver. The existence of a new case is signalized to the iPad user through the Apple Push Notification service. After login to the Aycan mobile App some meta data and thumbnails of the case are retrieved by the iPad. If both devices (Aycan mobile and Aycan OsiriX PRO) are logged in at the same network, they will establish a secure, encrypted channel and send the images directly to the iPad. If they are in different networks without routing, they will establish a secure, encrypted ad-hoc SSL tunnel between the devices and send the images for remote consultation on the iPad (Figure 1) [20,21].

**Figure 2:**
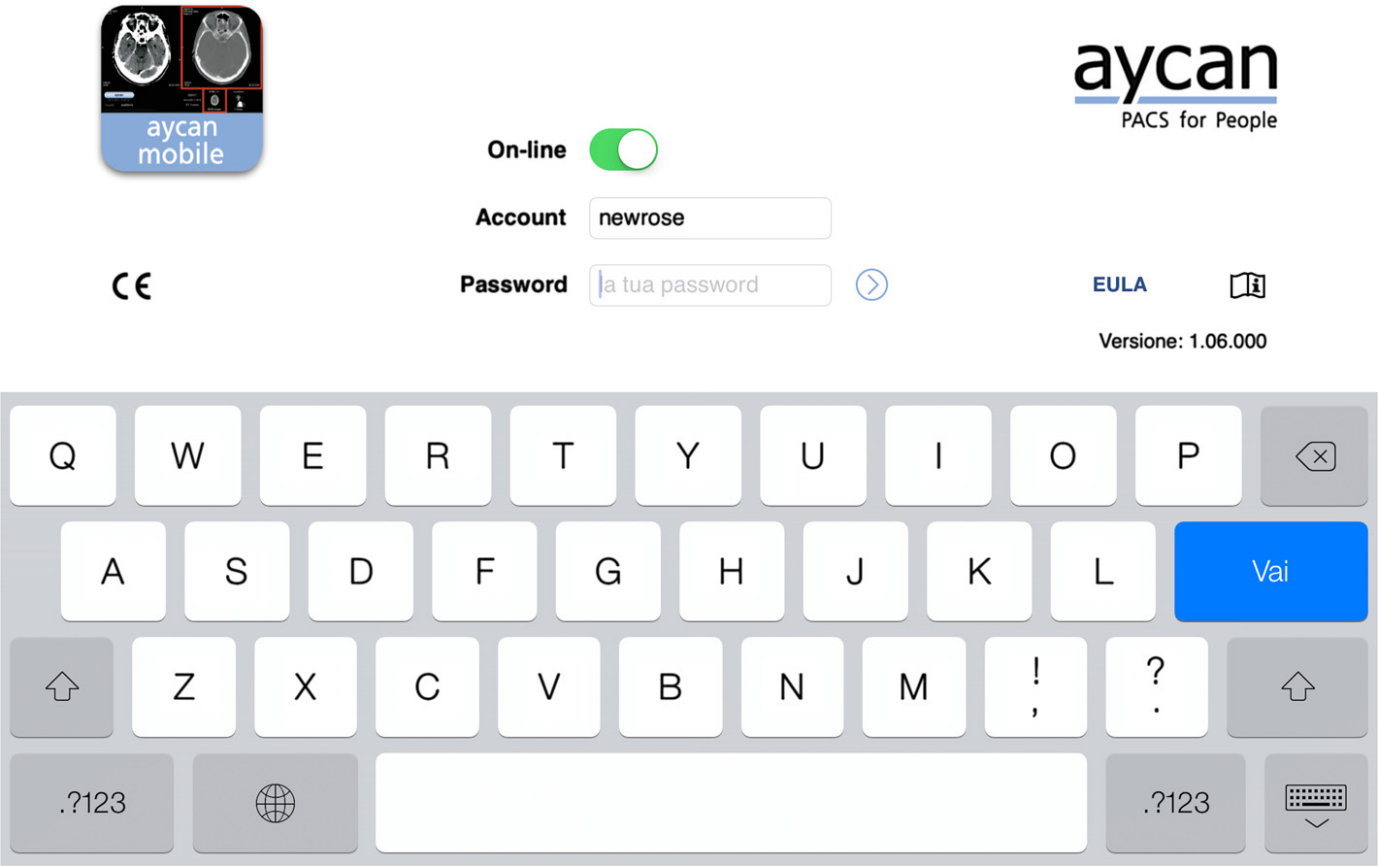
**Data access on iPad.** Data access is secured by a password to protect confidentiality.

**Figure 3:**
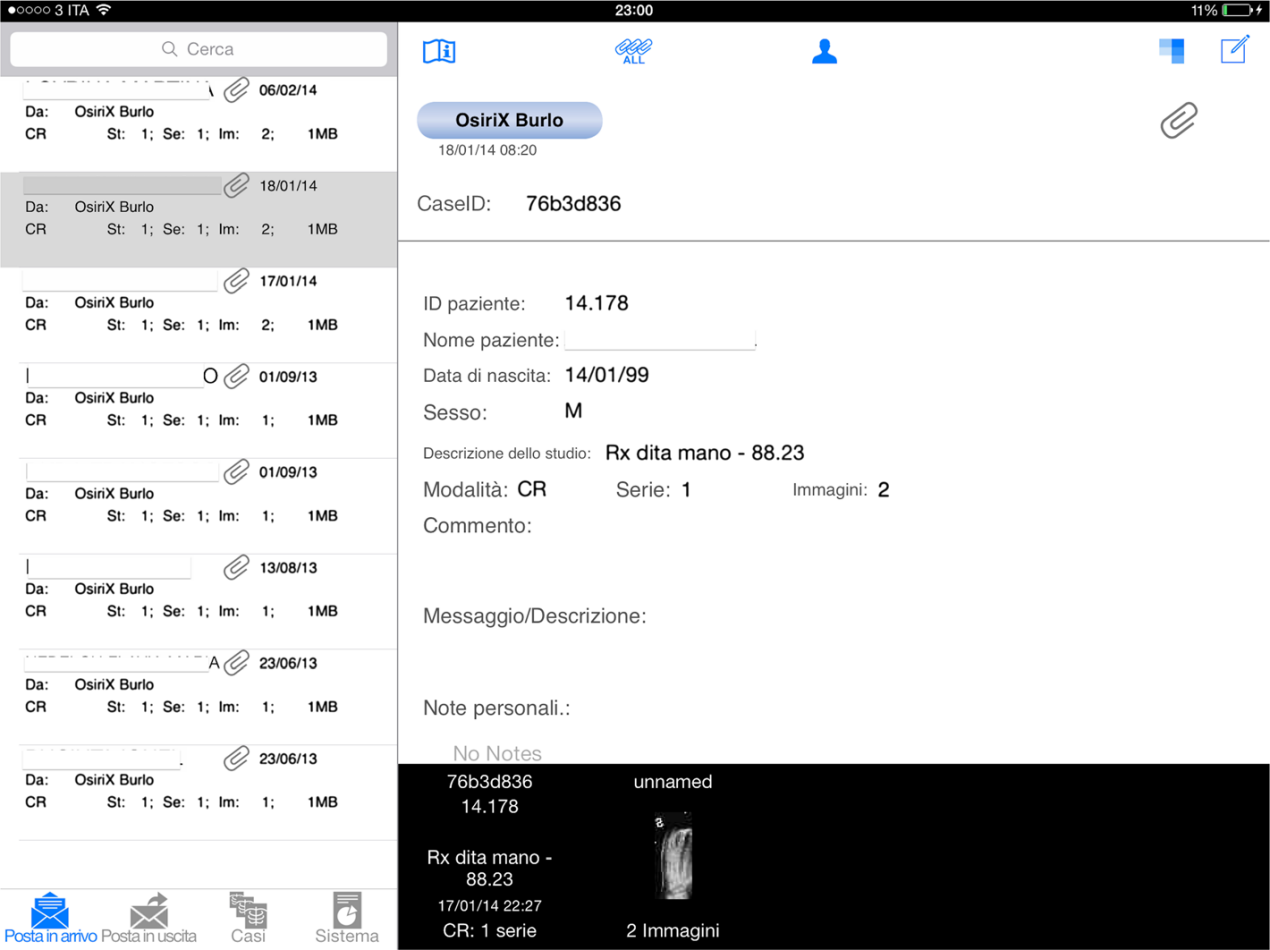
**Data transfer.** The patient name is not recorded by the system to protect confidentiality.

Confidentiality is ensured by the following mechanism: data is encrypted during transmission; data is stored encrypted on the device; data access is secured by a password (Figure 2); anonymization of the 'patient name' can be used by default or switched on/off for each individual transfer (Figure 3) [20,21].

To ensure accountability (i.e. that the prescribed access process is being done by an authorized user) the following steps are followed: every person utilising the system has to have a valid user account with an unique user name; people who would like to exchange images have to authorized each other before one is able to send data to the other; each image sent has an unambiguous sender and an unambiguous user as the addressee(s) of the message [20,21].

Data integrity is ensured by the following mechanisms: the data is encrypted during transmission (data modification would imply correct decryption and correct encryption after modification, which is not easily possible); incomplete or modified transmissions can be detected and notified; currently there are no known viruses and other compromising software affecting iOS [20,21].

The technology was implemented by a team composed by a radiologist (FZ), and a clinical engineer.

### Training

All eight technicians of the radiology department were trained by a radiologist (FZ) in using Aycan OsiriX PRO with a single session of 1 hour. Five orthopaedics specialists were trained in using the iPad and Aycan through a half hour session. There was no need to train paediatricians in the emergency department since images were sent directly from the radiological department to the orthopaedics on-call.

### Patients and study design

Patients were recruited in the Emergency paediatric unit of IRCCS Burlo Garofolo during September 2013 and October 2013. Children from 0 to 18 years, diagnosed with a bone fracture by the radiologist during the hours when orthopaedic service is provided only on-call (4 pm-8 am Monday to Friday, and from Saturday 1 pm to Monday 8 am), were eligible for enrollment.

Cases were enrolled prospectively. Selection of cases was done quasi-randomly, i.e. when the iPad device was available and not utilised by other studies. When the iPad was not available for the consultant on-call, cases were excluded from this study.

For each child the orthopaedic specialist received two sets of information, at subsequent times (Phase I and Phase II). Phase I was according to standard care (i.e. the paediatrician read the radiological report to the orthopaedic on-call, by phone) and the orthopaedic specialist recorded in a database his/her decision on case management based on this phone information, blinded to Phase II. As soon the decision from the consultant was recorded, Phase II started: the orthopaedic specialist received as additional information the X-ray for remote consultation on the iPad, and he/she recorded his/her decision in the database. For each clinical case, we compared decision of the orthopaedic specialist taken during Phase I (i.e. standard approach: only verbal communication, no X-ray provided) to decision taken during Phase II (standard approach *plus* X-ray provided for remote consultation through the iPad).

### Outcomes

The primary outcome of the study was the decision of the orthopaedic specialist who was on-call (i.e. outside the hospital) to perform an immediate in-hospital orthopaedic consultancy (i.e. the decision to come to the hospital to assess the patient).

Other outcomes of the study included: immediate activation of other services (such as the operating theatre and plaster room); time for the orthopaedic specialist to decide case-treatment; quality of images; diagnostic confidence; and number of technical difficulties.

Overall time for the orthopaedic specialist to decide case-treatment was calculated for Phase I from when the paediatrician finished reading the radiological report to the orthopaedic on call, to when the orthopaedic consultant expressed to the paediatrician his/her advice on case treatment. For Phase II time was calculated from when the X-ray image was sent to the orthopaedic to when the orthopaedic consultant expressed to the paediatrician his/her advice on case treatment. For cases in Phase I which the orthopaedic specialist decided than he/she needed to come to the hospital to see the X-ray, time for travel to the hospital was recorded as a standard time of 45 minutes to avoid bias in the time calculation due to other factors affecting travel time such as road traffic etc.; time after visualisation of the X-ray was also summed up to calculate total time on case management.

Both the quality of images and diagnostic confidence using iPad were compared to using the dedicated PACS workstation and blindly evaluated for each image by three paediatric radiologists independent to the study project and unaware of the study results. PACS workstation (EBIT AET DICOMed Review Radio, version V4.1, with a monitor with a resolution of 1600x1200 dpi certified for medical use) was chosen for this evaluation as this is the system used for radiological diagnosis in the hospital. A Likert scale of 1 to 10 was used to judge both quality of images and of diagnostic confidence, with a value of 10 indicating excellent quality or excellent confidence.

### Statistical analyses

Our a priori hypothesis was that the rate of in-hospital consultancies would have dropped from 80% without a remote X-ray provided to 40% when the X-ray was provided via iPad; it was estimated that 46 children were needed to detect a significant difference with a power of 80% and significance of 0.05%.

We carried out a descriptive analysis of data regarding the children with bone fractures whose digital X-ray images were sent through teleradiology. We used means and ranges for numerical variables, frequencies and proportions for categorical variables. Paired categorical variables were compared using the Mc Nemar exact test. Paired continuous variables were analysed with the *t* test for paired data when normally distributed, and with a non parametrical method, the Wilcoxon Matched-Pairs Signed-Ranks Test for paired data, when not normally distributed. All statistical tests were 2-sided. Quality of images and diagnostic confidence were calculated as the average evaluation between the three external radiologists. The inter-observers agreement was analysed with kappa statistics (k), where a value of k > 0.6-0.8 represents substantial agreement.

## Results

Forty-two children were enrolled in the study. Characteristics of cases are described in Table 1. Among the forty-two enrolled children, 23 (54.7%) presented with a fracture of the upper limbs, 13 (30.9%) with a fracture in the lower limbs, 5 (11.9%) with a fracture in the clavicle, and one with a suspected fracture of the pelvis. Overall, 25 (59.5%) children were diagnosed with a simple fracture, 15 (35.7%) children with a wedged fracture, and 2 (4.7%) children with a complex (displaced) fracture.

**Table 1:** Characteristics of children enrolled in the study

**Variable of interest**		**N (%)**
Number of children enrolled	N (%)	42 (100)
Age		
- Years	Mean (±SD)	5.9 (4.2)
Sex		
- Female	N (%)	23 (54.7)
Male		19 (45.6)
Type of fracture		
- Simple	N (%)	25 (59.5)
- Wedge		15 (35.7)
Complex		2 (4.7)
Site of fracture		
- Upper Limbs	N (%)	23 (54.7)
- Lower limbs		13 (30.9)
- Clavicle		5 (11.9)
Pelvis		1 (2.3)

The number of in-hospital consultancies by the orthopaedic specialists was 32/42 (76.1%) when no X-ray was provided compared to 16/42 (38%) when the X-ray was provided through the iPad, p < 0.001 (Table 2). No significant differences were observed among different orthopaedic specialists (p = 0.1).

**Table 2:** Efficacy outcomes

	**Standard approach (only verbal communication)**	**Standard approach *plus *** **X-ray through iPad**	**P value**
In-hospital orthopaedic consultations required	32 (76.1%)	16 (38%)	P < 0.001
Immediate activation of other services (surgery and plaster room)	0 (0%)	14 (33.3%)	P < 0.001
Average time for decision making on case treatment (in minutes)	56.2 ± 16.1*	23.4 ± 21.8	P < 0.001

*Including travel time to the hospital to view the x-ray if needed.

With teleradiology in 14/42 (33.3%) cases services such as surgery and plaster room could be immediately activated for case management, compared to no service immediately activated without teleradiology (p < 0.001).

**Figure 4:**
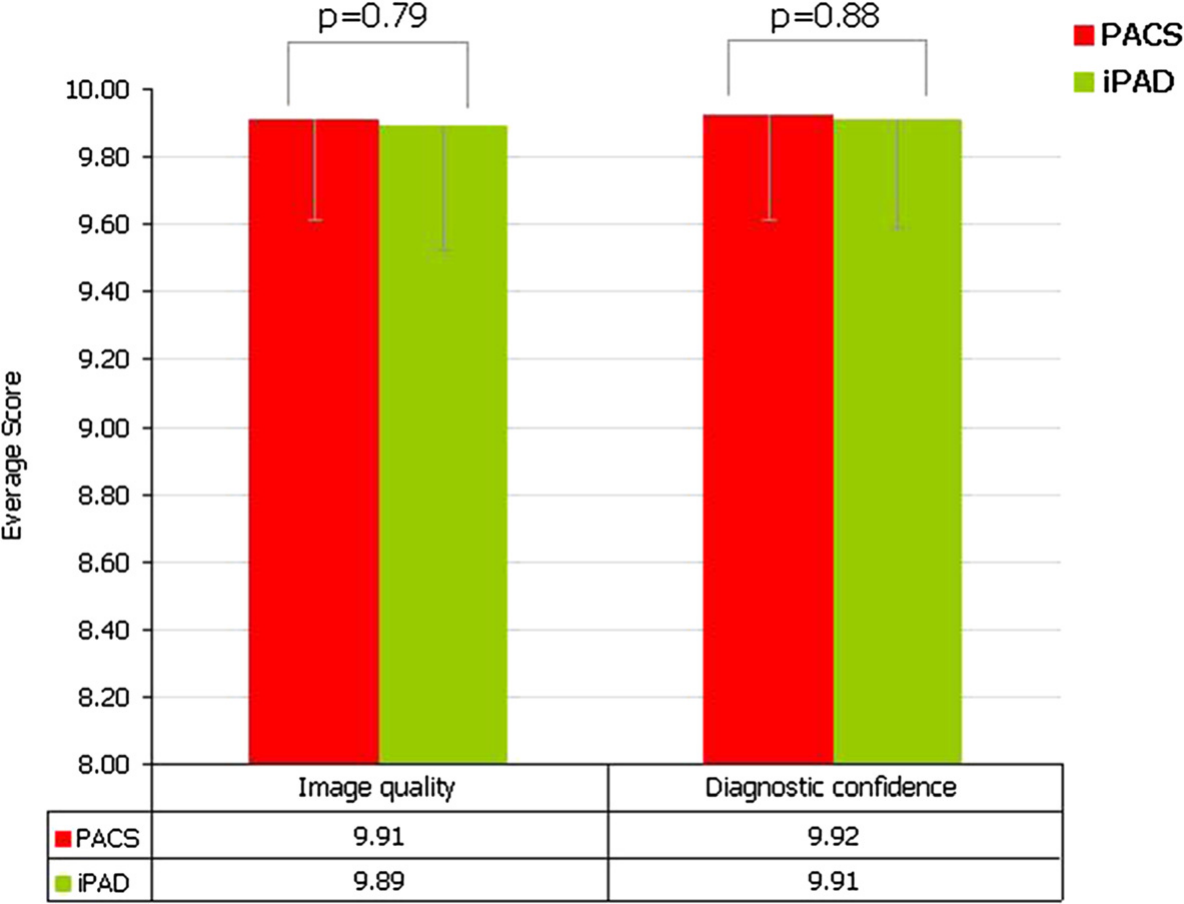
**Quality of images and diagnostic confidence.** There was not significant difference among quality of images and diagnostic confidence using PACS (green columns) and using iPad (in red columns).

With the standard approach (only verbal communication, no X-ray provided), the average time for decision making on case-management of fractures was 56.2 ± 16.1 minutes compared to 23.4 ± 21.8 minutes when the X-ray was provided (p < 0.001).There was no statistically significant difference in quality of images using iPad compared to the dedicated PACS, as rated by the independent radiologists (respectively 9.89 ± 0.37 K = 0.92 vs 9.91 ± 0.30 K0 = .96; p =0.79 (Figure 4). Similarly, there was no statistically significant difference in diagnostic confidence using the iPad compared to using the PACS (respectively 9.91 ± 0.32 K = 0.95 vs 9.92 ± 0.31 K = 0.92; p = 0.88).

## Discussion

This study was a pragmatic evaluation of the impact on in-hospital orthopaedic consultancies of providing the X-ray for remote consultation through an iPad in addition to the standard approach (verbal communication only). Results showed that after having direct access to the X-ray for remote consultation orthopaedics switched their minds from "go to the hospital" to "stay home", halving the number of in-hospital orthopaedic consultancies for paediatric fractures, and significantly speeding up the decision making on case management.

This study suggests that providing access to the X-ray for remote orthopaedics consultation may have a large impact on the organisation of care, decreasing the need for the orthopaedic to come to the hospital, and facilitating a more rational and efficient use of the health system resources. Potentially, although this was not formally evaluated in our research, it may also decrease the costs for care. Specialist orthopaedics are paid more for their on-call consultancy if they come to the hospital rather than if they work from home. In our setting, net cost for the in-hospital orthopaedic consultancy is 28.54 euro/hour (with an average of approximately 3500 consultancies per year, and an average duration of each consultancy of 1.8 hours, range from 30 minutes to 6 hours), whereas if the on-call orthopaedic provides the consultation only from home the cost is approximately 20 euro for the whole shift (usually 12 hours). Therefore the estimated total difference in expenditure for the hospital when implementing simple systems for remote X-ray consultations may add up to several thousand of euro a year.

Additionally, the recent economical crisis has imposed restrictions in the health workforces [1,22,23]. There are fewer orthopaedic specialists and these frequently need to cover the services on-call at night or during the weekend. In many instances even if called at night, the specialist is not exempted from working on the following day. Requiring a specialist to come back to the hospital just to view an X-ray, because this is not available for remote consultation, may appear outdated compared to the actual large use of web-based services in the general population.

Although teleradiology is a very rapidly growing field, to the best of our knowledge, there are no other studies in orthopaedics on systems for remote X-ray consultation ensuring adherence to international standards on data privacy, image quality, and data integrity. As already described, most studies in orthopaedics have evaluated MMS. However, with MMS data security is not ensured (for example, with MMS data usually stored in an unprotected server of the provider) and data integrity is problematic (e.g. sharing files of large dimension is often difficult, and image resolution may be automatically lowered by the system) [15-17]. Other systems for data transfer, such as Dropbox or email are regarded as potentially unsecure if they are not built on a dedicated system. The vision of the European *eHealth Action Plan 2012-2020*[1] is to utilise and develop eHealth to address several of the most pressing health and health systems challenges of the first half of the 21st century, including the need to increase sustainability and efficiency of health systems. This study aimed at filling gap in research in regards to innovative simple eHealth systems for orthopaedic consultants. A possible limitations of this study include the cost of the technology used. In the future, other systems may be tested and compared to Aycan OsiriX PRO or iPad, evaluating both accuracy in diagnosis and cost. For example, future systems for remote X-ray consultation may utilise smart phones with large screens, which are sometimes preferred to an iPad due to their portability.

## Conclusions

Remote X-ray consultation through an iPad should be considered as a means to reduce the need for in-hospital orthopaedic consultation, and potentially decrease costs for the health system and families. In the future, alternative systems less expensive than Aycan OsiriX PRO should be further developed and tested.

## Competing interests

The authors declare that they have no competing interests.

## Authors’ contributions

FZ conceived this study together with ML. DG, RF, MM, LO, VD, DD, FV, MB participated in the conduction of the study. ML wrote the first draft of this report; all authors revised the draft of the report and approved its final version.

## Pre-publication history

The pre-publication history for this paper can be accessed here:

http://www.biomedcentral.com/1472-6963/14/327/prepub
